# Assessment of Public Knowledge of Hypertension Among the Saudi Population

**DOI:** 10.7759/cureus.37649

**Published:** 2023-04-16

**Authors:** Sulaiman A Alshammari, Almaha H Alshathri, Aljohara H Alshathri, Sarah A Aleban, Durrah W Alabdullah, Jana K Abukhlaled, Sarah S Aldharman

**Affiliations:** 1 Family and Community Medicine, College of Medicine, King Saud University, Riyadh, SAU; 2 College of Medicine, King Saud University, Riyadh, SAU; 3 College of Medicine, Princess Nourah Bint Abdulrahman University, Riyadh, SAU; 4 College of Medicine, King Saud Bin Abdulaziz University for Health Sciences, Riyadh, SAU

**Keywords:** saudi arabia, knowledge, high blood pressure, cardiovascular, hypertension

## Abstract

Background

Hypertension is a major risk factor for cardiovascular illness and premature death and is becoming more prevalent worldwide. To promote better educational strategies regarding hypertension, it is crucial to identify the most significant knowledge gaps among the general public. This study aimed to assess the knowledge of hypertension among the general public in Saudi Arabia.

Methodology

A cross-sectional, questionnaire-based study was conducted in Saudi Arabia. The target population was the general public aged ≥18 in Saudi Arabia. Statistical analysis was conducted using RStudio (R version 4.1.1). Numerical data were described as mean ± standard deviation or median and interquartile range (IQR) whenever applicable. P-values <0.05 indicated statistical significance.

Results

A total of 1,404 respondents were collected. After exclusion, 1,399 records were analyzed in this study. More than half of the respondents were females (59.5%) aged 18-39 years (52.7%) and had a university degree (64.8%). Additionally, 46.0% were employed. Approximately one-quarter of the sample had hypertension (26.3%), while 73.3% had a family history of hypertension The median score was 16.0 (IQR = 12.0-18.0) with a minimum and a maximum of 0.0 and 22.0, respectively. Reliability testing revealed that knowledge items had a good internal consistency (Cronbach’s alpha = 0.859 based on 22 knowledge items). There was no significant association between knowledge and gender and having a personal history of hypertension. However, the knowledge score differed significantly by age, educational level, employment status, and having a family history of hypertension. On the multivariate analysis, knowledge scores were independently higher among participants in the higher age categories. Moreover, having a university degree, a postgraduate degree, and a family history of hypertension were independently associated with higher knowledge scores.

Conclusions

This study found that the general public in Saudi Arabia had good levels of knowledge about hypertension. Being knowledgeable about hypertension not only improves adherence to treatment plans among antihypertensive patients but also aids in avoiding its occurrence and consequences among non-hypertensive patients by adopting self-care. Serial and frequent studies on this issue are recommended to gather more evidence on this topic. Ongoing hypertension education is essential to enhance knowledge to minimize the burden of this prevalent issue.

## Introduction

Hypertension is an important health issue with a significant burden. It is defined as a systolic blood pressure greater than 140 mmHg and a diastolic blood pressure greater than 90 mmHg [1]. It is the most important cardiovascular risk factor and is regarded as a significant risk factor for serious cardiovascular disease, renal disease, aneurysms, peripheral artery disease, and strokes [1-3]. It has been referred to as a silent killer in numerous studies to draw attention to its severity [4]. Hypertension ranks among the leading factors in morbidity and mortality.

According to the World Health Organization (WHO) [1,2], hypertension affects 7 million people worldwide annually. In addition, the WHO reported that 1.28 billion adults worldwide, most of whom reside in low- and middle-income nations, suffered from hypertension. Adults with hypertension are only diagnosed and treated in 42% of cases. Only 21% of those with hypertension have it under control [5]. Worldwide, high blood pressure is a significant factor in premature death. One of the global goals for noncommunicable diseases such as hypertension is to reduce hypertension prevalence by 33% between 2010 and 2030 [5]. According to a global analysis, the number of people with uncontrolled hypertension (>140/90 mmHg) increased from 600 million in 1980 to almost 1 billion in 2008, and it is predicted that this number will rise to 1.56 billion by 2025, meaning that 29% of the adult population will have hypertension [6].

Previous studies in Saudi Arabia revealed that hypertension is increasing in prevalence, affecting more than a quarter of the Saudi population. This increase is mainly due to lifestyle changes, unhealthy diets, and obesity [6]. A previous local Saudi study conducted in Riyadh aimed to evaluate the awareness of hypertension and health-related quality of life among the hypertensive population [1]. It was found that the majority of the participants were aware of normal blood pressure levels. Furthermore, the knowledge of hypertension was significantly associated with quality of life. According to national surveys, many hypertensive patients are unaware of their disease, and the majority of those who are aware are not on treatment [7]. Treatment of hypertension predominantly relies on the patient’s perception of hypertension, level of understanding, and the magnitude of associated comorbidities [3]. A lack of knowledge about hypertension can lead to a greater burden on the healthcare system in Saudi Arabia with its increased prevalence [6,8]. This study aimed to assess public knowledge about hypertension among the population in Saudi Arabia.

## Materials and methods

This analytical, cross-sectional, questionnaire-based study was conducted in Saudi Arabia between August 2022 and February 2023. The target subjects were the general population of Saudi Arabia aged ≥18 from different regions (central, southern, eastern, western, and northern). The study was conducted through an online self-administered questionnaire. The questionnaire was distributed on different online platforms to enable data collection from different regions of Saudi Arabia. All responses were collected and exported into a Microsoft Excel spreadsheet file by Google Docs tools for processing and analysis.

Sample size and convenience sampling technique

Using OpenEpi® version 3.0 (Centers for Disease Control and Prevention (CDC), Atlanta, GA, USA), the sample size required was 385, with a margin of error of 5%, a confidence level of 95%, and a population size of 34 million, which is the Saudi population.

Inclusion and exclusion criteria

The study’s eligibility criteria included hypertensive patients and non-hypertensive individuals aged ≥18 years who were able to read and understand Arabic. Patients who declined to participate in the study and who were unable to read Arabic were excluded from this study.

Data collection instrument and procedure

The Arabic version of the Hypertension Knowledge-Level Scale (HK-LS) tool was obtained from a previous study [9]. The Arabic version of the HK-LS was found to be a reliable and valid tool for measuring knowledge about hypertension among the general population [10]. The questionnaire contains two main sections. The first includes sociodemographic characteristics such as age, gender, education level, employment, and personal and family history of hypertension. The second section contains items of the HK-LS tool. The HK-LS contains 22 items with six sub-dimensions, namely, definition, medical treatment, drug compliance, lifestyle, diet, and complications. The responses were encoded as yes, no, and don’t know. Each correct answer added to the total score of 1 point, whereas each incorrect and don’t know answer took no points. The maximum score was 22; as for the sub-dimensions, 2 points were for definition, 4 for medical treatment, 4 for drug compliance, 5 for lifestyle, 2 for diet, and 5 for the complications sub-dimension. The minimum score was 0 for both the entire scale and all sub-dimensions. The higher the score the higher the level of knowledge. The study was approved by the Institutional Review Board (IRB), Health Sciences Colleges Research on Human Subjects King Saud University, College of Medicine (project number: E-23-7533).

Statistical analysis

Statistical analysis was performed using RStudio (R version 4.1.1). Data were described as frequencies and percentages for categorical data. Based on the normality testing, numerical data were described as mean ± standard deviation (SD) or median and interquartile range (IQR) whenever applicable. Factors associated with the knowledge score were assessed using the Mann-Whitney test and the Kruskal-Wallis rank sum test for categorical variables with two categories or three or more categories, respectively. The significantly associated factors were further used as independent variables in a multivariate linear regression model to assess the independent predictors of knowledge among the participants. The outcomes of the regression analysis were presented as beta coefficients and their respective 95% confidence intervals (95% CIs). A p-value <0.05 indicated statistical significance.

## Results

Sociodemographic characteristics

A total of 1,404 responses were collected on the online platform. However, we excluded five responses from those who declined to participate. Therefore, 1,399 records were analyzed in this study. More than half of the respondents were females (59.5%) aged 18-39 years (52.7%) and had a university degree (64.8%). Additionally, 46.0% of them were employed. Approximately one-quarter of the sample had hypertension (26.3%), whereas 73.3% had a family history of hypertension (Table 1).

**Table 1:** Sociodemographic characteristics of the participants.

Parameter	Category	N (%)
Gender	Male	567 (40.5%)
	Female	832 (59.5%)
Age (years)	18–29	534 (38.2%)
	30–39	203 (14.5%)
	40–49	337 (24.1%)
	50–59	269 (19.2%)
	>60	56 (4.0%)
Educational level	General education	403 (28.8%)
	University	907 (64.8%)
	Postgraduate	89 (6.4%)
Employed	No	756 (54.0%)
	Yes	643 (46.0%)
Personal history of hypertension	No	1,031 (73.7%)
	Yes	368 (26.3%)
Family history of hypertension	No	373 (26.7%)
	Yes	1,026 (73.3%)

Participants’ responses to knowledge items

In general, 57.1% of the respondents correctly stated that hypertension can be indicated by increased systolic or diastolic blood pressure (57.1%), and 44.9% identified that only diastolic blood pressure can indicate hypertension. Great proportions of the participants indicated that hypertension medications should be taken daily (80.3%), taken always (56.9%), and should not be taken when they are tired (71.6%). Regarding the patterns of medication use, 55.0% of the participants indicated that patients should not limit taking the medications to when they are relaxed and that there is no need to not change the daily routine and behaviors if the hypertension is adequately controlled (66.5%). While the majority of respondents disagreed that there is no need to take medications because hypertension occurs with old age (73.9%), only 39.3% disagreed that there is no need to take medications if the hypertension patient succeeded to change his/her lifestyle and healthy behaviors in his/her life.

Almost two-thirds of the respondents declared that hypertensive patients cannot consume salty food only with taking hypertension medications, cannot consume alcohol (72.8%), should not smoke (68.9%), and should eat fruits and vegetables regularly (78.5%). Considerable proportions of the respondents correctly stated that fried food is the best method to prepare meals for hypertensive patients (73.3%) and that boiled and grilled food is not suitable for them (81.0%). More than half of the sample mentioned that white meat is the best type (57.7%) and red meat is not a good choice for patients with hypertension (50.4%). The majority of the respondents correctly identified that untreated hypertension can lead to early death (75.6%), cardiovascular disease (84.1%), stroke (80.8%), renal failure (61.0%), and eye and vision problems (78.3%) (Table 2).

**Table 2:** Participants’ responses to knowledge items. *An asterisk indicates a correct answer.

Parameter	False	True	Do not know
Only diastolic blood pressure is an indicator of hypertension	187 (13.4%)	628 (44.9%)*	584 (41.7%)
Increased systolic or diastolic blood pressure is an indicator of hypertension	102 (7.3%)	799 (57.1%)*	498 (35.6%)
The prescribed medications for hypertension should be taken daily	92 (6.6%)	1,124 (80.3%)*	183 (13.1%)
Hypertension patients should only take their medications when they are tired and not well	1,002 (71.6%)*	216 (15.4%)	181 (12.9%)
Hypertension patients should always take their medications	278 (19.9%)	796 (56.9%)*	325 (23.2%)
Hypertension patients should take their medications in a way in which they feel relaxed	769 (55.0%)*	387 (27.7%)	243 (17.4%)
There is no need to change the daily routine and behaviors of the patient if medications are able to control hypertension	931 (66.5%)*	255 (18.2%)	213 (15.2%)
Hypertension occurs with old age so no need to take medications in this condition	1,034 (73.9%)*	151 (10.8%)	214 (15.3%)
No need to take medications if the hypertension patient succeeded to change his/her lifestyle and healthy behaviors in his/her life	550 (39.3%)*	514 (36.7%)	335 (23.9%)
Hypertension patients can eat salty food only when they take their medications regularly	931 (66.5%)*	249 (17.8%)	219 (15.7%)
Hypertension patients can drink alcohol	1,019 (72.8%)*	116 (8.3%)	264 (18.9%)
Hypertension patients should not smoke	201 (14.4%)	964 (68.9%)*	234 (16.7%)
Hypertension patients should eat fruits and vegetables regularly	72 (5.1%)	1,098 (78.5%)*	229 (16.4%)
Frying the food is the best way to prepare meals for hypertension patients	1,025 (73.3%)*	151 (10.8%)	223 (15.9%)
Boiling and grilling the food are the best ways to prepare meals for hypertension patients	70 (5.0%)	1,133 (81.0%)*	196 (14.0%)
White meats are the best type of meat for hypertension patients	124 (8.9%)	807 (57.7%)*	468 (33.5%)
Red meats are the best type of meat for hypertension patients	705 (50.4%)*	212 (15.2%)	482 (34.5%)
Untreated hypertension can lead to early death	83 (5.9%)	1,057 (75.6%)*	259 (18.5%)
Untreated hypertension can cause cardiovascular diseases such as thrombi	51 (3.6%)	1,176 (84.1%)*	172 (12.3%)
Untreated hypertension can cause stroke	58 (4.1%)	1,130 (80.8%)*	211 (15.1%)
Untreated hypertension can cause renal failure	130 (9.3%)	854 (61.0%)*	415 (29.7%)
Untreated hypertension can cause eye and vision problems	64 (4.6%)	1,095 (78.3%)*	240 (17.2%)

Description of the knowledge score

**Figure 1:**
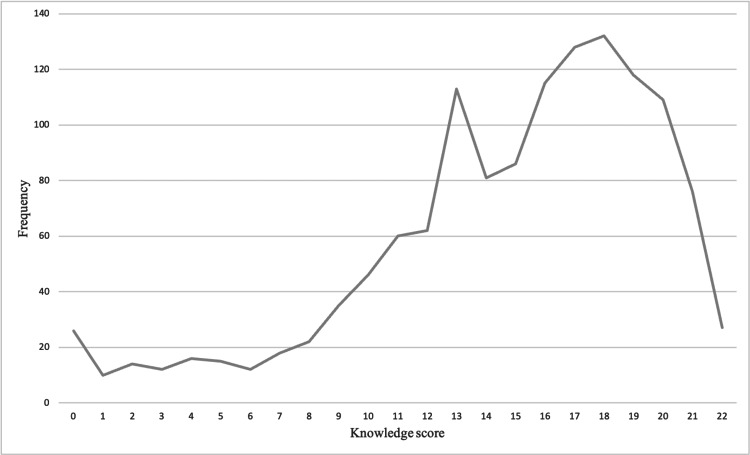
Curve diagram depicting the frequency distribution of the knowledge score.

Results of the normality testing showed that the knowledge score was non-normally distributed (Shapiro-Wilk test, p < 0.0001). The frequency distribution of the knowledge score is depicted in Figure 1. The median score was 16.0 (IQR = 12.0-18.0), with a minimum and a maximum of 0.0 and 22.0, respectively. Reliability testing revealed that knowledge items had a good internal consistency (Cronbach’s alpha = 0.859 based on 22 knowledge items).

Factors associated with knowledge

Based on the association analysis, there were no significant differences in the knowledge score in terms of gender and having a personal history of hypertension. However, the knowledge score differed significantly by participants’ ages (p < 0.001), educational levels (p < 0.001), employment status (p = 0.005), and family history of hypertension (p < 0.001) (Table 3).

**Table 3:** Factors associated with the knowledge score.

Parameter	Category	Median (IQR)	P-value
Gender	Male	16.0 (12.0, 19.0)	0.361
	Female	16.0 (12.0, 18.0)	
Age (years)	18–29	14.0 (11.0, 18.0)	<0.001
	30–39	15.0 (12.0, 18.0)	
	40–49	17.0 (13.0, 19.0)	
	50–59	17.0 (14.0, 19.0)	
	>60	16.0 (13.8, 19.0)	
Educational level	General education	15.0 (11.0, 18.0)	<0.001
	University	16.0 (13.0, 19.0)	
	Post-graduate	17.0 (13.0, 20.0)	
Employed	No	16.0 (12.0, 18.0)	0.005
	Yes	16.0 (13.0, 19.0)	
Personal history of hypertension	No	16.0 (12.0, 18.0)	0.513
	Yes	16.0 (13.0, 19.0)	
Family history of hypertension	No	13.0 (10.0, 17.0)	<0.001
	Yes	17.0 (13.0, 19.0)	

On the multivariate analysis, the scores of knowledge were independently higher among participants with higher age categories, including 40-49 years (beta = 1.53, 95% CI = 0.83 to 2.23, p < 0.001), 50-59 years (beta = 2.59, 95% CI = 1.88 to 3.30, p < 0.001), and >60 years (beta = 2.24, 95% CI = 0.94 to 3.54, p < 0.001). Additionally, having a university degree (beta = 1.75, 95% CI = 1.17 to 2.33, p < 0.001) and a postgraduate degree (beta = 3.29, 95% CI = 2.16 to 4.41, p < 0.001) were independently associated with higher knowledge scores. Having a family history of hypertension also predicted higher knowledge scores (beta = 2.83, 95% CI = 2.26 to 3.39, p < 0.001) (Table 4).

**Table 4:** Results of the multivariate regression analysis for the predictors of knowledge regarding hypertension.

Parameter	Category	Beta	95% CI	p-value
Age (years)	18–29	Ref	Ref	
	30–39	-0.10	-0.90, 0.71	0.814
	40–49	1.53	0.83, 2.23	<0.001
	50–59	2.59	1.88, 3.30	<0.001
	>60	2.24	0.94, 3.54	<0.001
Educational level	General education	Ref	Ref	
	University	1.75	1.17, 2.33	<0.001
	Postgraduate	3.29	2.16, 4.41	<0.001
Employed	No	Ref	Ref	
	Yes	-0.19	-0.76, 0.37	0.502
Family history of hypertension	No	Ref	Ref	
	Yes	2.83	2.26, 3.39	<0.001

## Discussion

Hypertension gradually and irreversibly damages target organs, resulting in potentially fatal consequences and mortality [9]. To our knowledge, this is the first study conducted in Saudi Arabia using the HK-LS. The goal of this study was to evaluate the level of knowledge among hypertension patients. Accordingly, both participants with and without hypertension in our study exhibited good general basic knowledge about hypertension, with more than half of the participants having good knowledge.

Previous studies have reported conflicting information regarding the awareness of hypertension among hypertensive patients. One study conducted in Pakistan revealed that 81.1% of hypertensive participants had insufficient general knowledge of hypertension, and they are unaware of the significance of high blood pressure values [11]. Increased understanding, treatment, and control of hypertension may help to lower the high rates of cardiovascular disease-related mortality [2].

Results from our study showed that knowledge scores differed significantly by participants’ ages. Scores were higher among participants in higher age categories. This study also revealed a statistically significant link between patient education and hypertension knowledge, in which a university and postgraduate degree were independently associated with higher knowledge scores. This was consistent with the studies by Oumar et al., Eshah et al., and Zinat Motlagh et al. [2,12,9]. However, another study by Lugo-Mata et al. is inconsistent with our findings [13].

Our study showed that people who were employed had significantly better knowledge than those who were not employed. This may be explained by higher income levels and education. Moreover, our study showed that those who had a family history of hypertension had significantly better knowledge than those who did not. This was similar to other studies conducted by Lugo-Mata et al. and Grad et al., who reported that participants with a family history of hypertension had a significantly higher level of knowledge compared to those without a family history of hypertension [13,14]. Also, a study was conducted to determine the knowledge and educational requirements among hypertensive patients [15]. Contradictory to our results, there was no significant association between knowledge and sociodemographic variables including age, educational status, and occupation [15]. However, it was reported that the overall knowledge of hypertensive patients was good [15].

About two-thirds of our respondents stated that hypertensive patients should not smoke or consume alcohol. Furthermore, they stated that hypertensive patients should not consume salty food unless they are also taking medicine to treat their condition. This is in line with a Saudi Arabian study in 2021 which found that most of the participants accurately recognized smoking, consuming fatty meals, and being overweight as risk factors for hypertension [6].

Our study revealed that the Saudi population had good knowledge of complications followed by a diet of hypertension. This finding is consistent with a Jordanian and Saudi study [6,9]. However, the lowest level of knowledge was regarding drug compliance. This finding was explained by a previous study that either due to hypertension being a silent disease, they thought to take it only when the symptoms start to appear or might be a misunderstanding of the statements, and our previous study suggested changing the statement for better understanding [10].

Study strengths and limitations

Despite using an online questionnaire, an adequate sample size was achieved. Additionally, a validated scoring system was used to assess the level of knowledge. However, this study is limited by its cross-sectional design, the self‐reported data, not subject to independent verification, and potentially influenced by recall bias. Recall bias refers to the systematic difference between the ability of participant groups to accurately recall information. We used the HK-LS tool to measure the knowledge. In this scale, some questions asked depended on whether the participant had previous exposure to the information (e.g., untreated hypertension can cause cardiovascular diseases such as thrombi). Therefore, this can be affected by recall bias.

## Conclusions

The knowledge level of hypertension among the Saudi population was good among both hypertensive and non-hypertensive individuals. Greater effort is recommended to improve the level of knowledge, especially about drug compliance and medical treatment, among the Saudi population to achieve better blood pressure control. Such an initiative would be strengthened by collaboration between the Saudi Ministry of Health, local health authorities, and the Saudi Hypertension Management Society. Factors associated with a higher level of knowledge included age, education level, employment status, and family history of hypertension. Being knowledgeable about these factors is crucial for providing education programs and adapting public policies.
